# The relationship between neuroticism and mobile phone use among college students in love: The masking effect of self-emotional assessment

**DOI:** 10.3389/fpsyg.2022.942520

**Published:** 2022-09-15

**Authors:** Weijing Chen, Xiaoqian Wang, Shan Sun, Qian Liu, Zhiwen Guo

**Affiliations:** ^1^Business School, Hubei University, Wuhan, China; ^2^Hubei Center for Studies of Human Capital Development Strategy and Policy, Key Research Base of Humanities and Social Science of Hubei Province, Wuhan, China; ^3^Department of Psychology, School of Education, Hubei University, Wuhan, China; ^4^Library, Central China Normal University, Wuhan, China

**Keywords:** neuroticism, mobile phone use, self-emotional assessment, love status, moderated mediation

## Abstract

The relationship between neuroticism and mobile phone use is a hot research topic in the academic community. The purpose of this study was to investigate the roles of self-emotional assessment and love status in the mechanism through which college students’ neuroticism influences mobile phone use.We construct a moderated mediation model, and taking 869 Chinese college students as the research object and testing the mediating role of self-emotional assessment and the moderating role of love status. The results show that: (1) neuroticism was significantly positively related to mobile phone use and significantly negatively related to self-emotional assessment; self-emotional assessment was significantly positively related to mobile phone use; (2) self-emotional assessment had a masking effect on the relationship between neuroticism and mobile phone use; (3) love status not only moderated the relationship between self-emotional assessment and mobile phone use but also moderated the process through which self-emotional assessment masked the effect of neuroticism on mobile phone use. Our research expands the literature on the mechanisms underlying the effects of neuroticism on mobile phone use, enriches the understanding of the pertinent boundary conditions, and provides a better explanatory basis for the mobile phone use of college students.

## Introduction

With the arrival of the information age, mobile phones have gradually become integral to people’s lives. According to the China Internet Network Information Center’s Statistical Report on China’s Internet Development (CNNIC, 2022), China’s Internet penetration rate as of December 2021 had reached 73%. This meant that there were about 10.32 billion Internet users in China among which mobile phone users accounted for 99.7%, and people aged 10–19 and 20–29 accounted for 13.3 and 17.3%, respectively. Students accounted for 29.6%, ranking first in terms of occupation among all Chinese Internet users. As a group of college students with rapid ideological development and easy to accept new things, mobile phones have a subtle impact on their growth and development (Mei et al., 2018; He et al., 2019; Xiong et al., 2022). For college students, mobile phone use is a double-edged sword. On the one hand, mobile phones provide college students with a private space and a platform for self-display. Through mobile phone use, individuals can establish and maintain a good social circle obtain social support (Zhang et al., 2021) and sense of control in communication (Volungis et al., 2020). On the other hand, prolonged mobile phone use may also bring about a lot of negative effects on college students physically and mentally such as anxiety and loneliness (Kumar and Anupama, 2019; Jacob, 2020; Liu et al., 2020b), sleep disorders (Ma et al., 2022), poor school performance (Gao et al., 2020) and so on. As a result, research on smartphone use is receiving increasing attention.

Among the many factors affecting mobile phone use, the influence mechanism of neuroticism on individual mobile phone use has always been an important research topic in academia. The majority of studies have based themselves on the theory of emotional dissonance, which suggests that high neurotic individuals are more often in a state of emotional instability (Brandes and Tackett, 2019; Xiong et al., 2022) and thus need to seek ways to cope with negative emotions more often (Zhang et al., 2018), mobile phone use satisfies their need to communicate with others and integrate into society, alleviates their negative emotions, and provides them with psychological compensation (Liu and Campbell, 2017; Carvalho et al., 2018; Lei et al., 2020; Bowden-Green et al., 2021).

In exploring the mechanism of “neuroticism–mobile phone use,” previous studies have demonstrated the mediating roles of coping style (Liu et al., 2020a), social anxiety (Jiang et al., 2020), metacognition (Marino et al., 2016), and subjective well-being (Zhang et al., 2020) in the relationship between neuroticism and mobile phone use. However, the role of emotion has often been ignored. As an exception, Zhang et al. (2017) focused on the mediating role of negative emotions in the relationship between neuroticism and mobile phone use. In their study, they focused on individual emotional experiences and feelings. We aimed to trace the causes potentially underlying the recognition, interpretation, and transformation of emotions. We focused on self-emotional assessment, which reflects the ability of individuals to consciously perceive, understand, and clearly express their emotions, and thus constitutes an important aspect of emotional intelligence (Salovey and Mayer, 1997). Whether an individual can correctly understand and evaluate their emotions affects to some extent their subsequent emotional experience, and whether they can make good use of external channels and tools (such as mobile phones) to express and regulate their emotions. Our study aimed to investigate the psychological processing mechanism by which neuroticism influences mobile phone use from the perspective of self-emotional assessment.

Being in love is often an important experience for college students. But fewer research has been done on whether and how this experience affects their mobile phone use Mobile phone use increases individuals’ satisfaction with their romantic relationships (Kaiser, 2018). Compared with single college students，college student couples may also interact more with each other using social media software on their mobile phones when they are apart. Meanwhile, individuals in love experience less alexithymia and are more likely to perceive and express emotions (Yu, 2021). Whether this period will affect their mobile phone use and the influencing mechanism have not been studied yet, which is also what we hope to clarify in the study.

In summary, our research explored the roles of self-emotional assessment and love status in the mechanism by which neuroticism influences mobile phone use among college students. We expanded the research illustrating the effects of neuroticism on mobile phone use and explored the boundary conditions delimiting these effects. In addition, we aimed to provide a deeper understanding of the formation of college students’ mobile phone use habits and a reference for researchers hoping to predict students’ mobile phone use or implement interventions.

## Literature review and hypotheses

The concept of neuroticism was first introduced by the psychologist Eysenck in his theory of personality in opposition to emotional stability (Eysenck and Himmelweit, 1946). People with a high tendency to experience neuroticism are typically described as having a neurotic personality (McCrae and Sutin, 2018). Individuals with highly neurotic personality traits often experience negative psychological conditions such as anxiety, stress, and depression, and display large mood fluctuations, strong responses to stimuli, and sometimes even irrational behaviors (Brandes and Tackett, 2019; Friedman, 2019; Xiong et al., 2022).

The positive correlation between neuroticism and mobile phone use has been supported by many empirical studies (Liu and Campbell, 2017; Carvalho et al., 2018; Lei et al., 2020; Bowden-Green et al., 2021). Because highly neurotic individuals are more likely to experience negative emotions such as stress and anxiety, they spend more time using mobile phones and their Internet functions to relieve these negative emotions than non-highly neurotic individuals. Highly neurotic people may be more likely to hide their concerns in the online social networking communication, because social anxiety, sensitive to criticism of others and reckless behavior may not be obvious in the virtual interaction, and the Internet environment can provide a safe area, make the neurotic individuals have enough sense of security to express themselves, thus reducing the pain associated with real social conditions (Boyes and French, 2012; Marriott and Buchanan, 2014). A systematic review (Bowden-Green et al., 2021) and a meta-analysis (Marciano et al., 2020) also further confirmed the positive relationship between neuroticism and mobile phone use, finding that neurotic individuals are not motivated to expand their social ties, it is about using online social media to reduce the pain associated with real social situations.

The social compensation hypothesis holds that people who have difficulties in face-to-face offline communication will make up for the lack of offline communication through online means (Kraut et al., 2002; Gross et al., 2010), Therefore, based on the social compensation hypothesis, when neurotic individuals experience a high level of anxiety during in-person social interactions, their feelings of deficiency predispose them to escape from in-person social interactions and instead meet their psychological needs through social apps or mobile games on their phones (Grieve et al., 2017; Evren et al., 2019; Lesmana et al., 2020; Marciano et al., 2020).

Among the many factors influencing mobile phone use, emotional factors play an important role (Gao and Chen, 2006; Volungis et al., 2020). However, they have often been overlooked in studies investigating the mechanism by which neuroticism influences mobile phone use. Zhang et al. (2017) have investigated the mediating role of negative emotions from the perspective of emotional experience, which only focuses on the emotional experience itself, while the explanation of its generation and cognitive process is equally important. But whether an individual can correctly understand and evaluate their emotions affects to some extent their subsequent emotional experience, and whether they can make good use of external channels and tools (such as mobile phones) to express and regulate their emotions. However, prior to our study, no researchers had explored the relationship between neuroticism and mobile phone use from the perspective of emotional cognition or the generation of emotional experience.

### The masking effect of self-emotional assessment

#### Neuroticism and self-emotional assessment

Self-emotional assessment refers to the ability of individuals to consciously perceive, understand, and clearly express their emotional states. Individuals with outstanding ability in this respect are more likely to correctly assess their emotions (Mayer and Geher, 1996; Salovey and Mayer, 1997). As one of the personality traits most closely linked with emotions, neuroticism is often associated with characteristics such as emotionality, impulsivity, and anxiety (Eysenck and Himmelweit, 1946). Jiang and Yang (2006) showed that emotional sensitivity plays an important mediating role in the process through which personality influences emotional regulation and self-efficacy. Therefore, personality traits may also interact with self-emotional assessment ability.

According to the complaint hypothesis of cognitive failure, individuals with high neuroticism are excessively obsessed with self-attention and experience more dysfunction in self-awareness attributes that manifest in the form of unreasonable complaints or worries about objective existence and cognition-related matters, therefore, they experience more self-reported cognitive failure (Schoo et al., 2013) In addition, neuropsychological models suggest that individuals with higher neuroticism experience higher levels of non-specific arousal (Arnett and Newman, 2000), which leads to a large proportion of their cognitive resources, such as their sensory and perceptual attention, being occupied by stimuli. Such individuals thus fail to self-regulate (Koenen and Karbach, 2020; Zhang et al., 2020; Pu et al., 2021) and may also experience a decline in their self-emotional assessment ability (Wallace et al., 1991). Therefore, we proposed the following hypothesis:

*Hypothesis 1*: Neuroticism is negatively related to self-emotional assessment.

#### The mediating role of self-emotional assessment

Individuals with high neuroticism tend to have low levels of self-emotional assessment, which in turn affects their use of mobile phones. Self-emotional assessment not only measures an individual’s ability to pay attention to and perceive their own emotions but also examines their ability to understand their own emotions (Mayer and Geher, 1996; Salovey and Mayer, 1997). According to the cognitive-behavioral model (Davis, 2001) individuals with high levels of self-emotional assessment can more accurately perceive their emotions, and their emotional cognition will further affect their behaviors (such as expressing emotions through external channels). As the socializing tool used the most by college students, the mobile phone has its unique advantages and is an important channel through which individuals can express their emotions. Gao and Chen (2006) found that individuals who are addicted to mobile phones have much more sensitive emotional perceptions than others. Beranuy et al. (2009) also demonstrated that the more attention individuals pay to emotions, the higher the degree of their mobile phone use. Therefore, individuals who make more accurate self-emotional assessments and have a better understanding of their own emotions are more likely to seek suitable ways (such as mobile phones) to convey and express their emotions or solve problems.

Previous studies have shown that individuals with high neuroticism increase the negative impact of risk factors (stressful life events) through cognitive processes (Ormel et al., 2013). Self-emotional assessment, as a process and important experience for individuals to recognize and understand their emotions, may play an important role in bridging the relationship between neuroticism and mobile phone use. Therefore, we predicted that individuals with high neuroticism would reduce their use of mobile phone through the self-emotional assessment process; in other words, the low self-emotional assessment levels associated with high neuroticism masks some of the effects of high neuroticism on mobile phone use. Therefore, we proposed the following hypotheses:

*Hypothesis 2*: Self-emotional assessment is positively related to mobile phone use.

*Hypothesis 3*: Self-emotional assessment masks the effect of neuroticism on mobile phone use.

### The moderating role of love status

In addition to personality, which is relatively stable, mobile phone use may also be influenced by more changeable emotional factors in life such as love status. With the development of technology, mobile phones have become a widely used communication medium. As mobile phones can allow people to share and communicate anytime and anywhere, thus enhancing intimacy, they are the primary medium through which young couples carry out their communications (Hsu et al., 2011). College students who are in love tend to have volatile moods (Yu, 2021). However, individuals’ decoding and cognition of their own emotions will affect their subsequent decisions and actions. Therefore, for individuals with large mood fluctuations, the level of self-emotional assessment is particularly important. The high level of self-emotonal assessment ability enables them to better assess their emotions and choose a more appropriate way to express their emotions and solve problems. Therefore, we predicted that for students in love, the level of self-emotional assessment has a greater impact on their decisions and behaviors than for students not in love. In other words, self-emotional assessment has a more significant predictive effect on mobile phone use for students who are in love.

Consequently, to explore the moderating effect of love status on the relationship between self-emotional assessment and mobile phone use, as well as its moderating effect on the process through which self-emotional assessment masks the effect of neuroticism on mobile phone use, we proposed the following hypotheses:

*Hypothesis 4*: Love status moderates the relationship between self-emotional assessment and mobile phone use such that the relationship is more positive when the student is in love.

*Hypothesis 5*: Love status moderates the process through which self-emotional assessment masks the effect of neuroticism on mobile phone use; that is, for the student who is in love, this masking effect is stronger.

**Figure 1:**
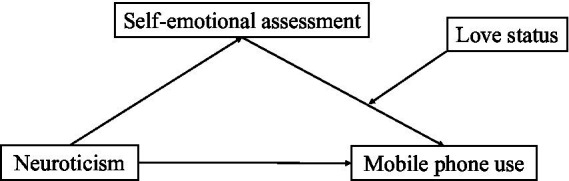
Research model.

Figure 1 depicts the research model of this study.

## Materials and methods

### Participants and procedures

We used the convenience sampling method to recruit participants *via* social media. Participants came from universities in Chinese cities (Shanghai, Beijing, Guangzhou, Wuhan, etc.), including college students, undergraduate students, master’s students, and doctoral students. We collected data by issuing electronic questionnaires online. Due to the setup of the questionnaire system only when all the questions have been answered can participants submit them successfully, therefore, there was no data missing. However, 52 subjects who chose the same option repeatedly and answered within 300 s were regarded as invalid and deleted. In this survey, nine hundred twenty-one questionnaires were distributed and all of the questionnaires were collected, of which 869 were deemed valid (effective response rate: 86.25%).

Among the 896 valid respondents, 31.3% were male and 68.7% were female, age range from 18 to 26; 65.8% were not in love and 34.2% were in love. With respect to education background, 83.8% had undergraduate degree level and 16.2% had master’s degree level and above. Moreover, with regard to the place of origin, 58.6% were from countryside, 46.8% were from urban areas, and 41.4% were the only child in their family, the other 58.6% were not.

### Measures

#### Neuroticism

Neuroticism was measured using a 24-item scale adapted from the neuroticism subscale in the Eysenck Personality Questionnaire (Eysenck, 1946) revised by Chen (1983). A sample item is “Do you have frequent mood swings?” Each item was scored on a 2-point scale (1 = Yes, 0 = No). Cronbach’s alpha was 0.823.

#### Mobile phone use

Mobile phone use was measured using the 17-item scale devised by Wilska (2003) for a questionnaire on the mobile phone use style of college students. As all our participants were Chinese, we followed the double-blind backtranslation procedure (Schaffer and Riordan, 2003) to translate all items into Chinese. A sample item is “It’s important for me to receive a lot of phone calls and messages.” Each item was scored on a 5-point Likert scale (1 = *strongly disagree*, 5 = *strongly agree*). Cronbach’s alpha was 0.856.

We further performed confirmatory factor analysis to validate the mobile phone use’ convergent validity and discriminant validity and obtained modest fit indices: *χ*^2^(87) = 240.287, *χ*^2^/df < 3, CFI = 0.921, NNFI = 0.905,RMSEA = 0.054, which reached the mediocre fitting level (Bentler et al., 1980). In addition, the factor-loading of most of the items are greater than 0.5; only two of them are just below 0.5. In terms of Bagozzi et al. (1999)’s suggested criteria (AVE ≥ 0.50), the AVE value of the mobile phone use is 0.57, which is acceptable.

#### Self-emotional assessment

Self-emotional assessment was measured using the 4-item self-emotional assessment subscale from the Chinese version of the emotional intelligence scale (WLELS-C) revised by Law et al. (2004). A sample item is “I can usually tell why I’m feeling certain things.” Each item was scored on a 5-point Likert scale (1 = *strongly disagree*, 5 = *strongly agree*). Cronbach’s alpha was 0.878.

#### Control variables

We controlled for gender, age, place of origin, and only child status. In addition, we controlled for extraversion, psychoticism, and the time taken by individuals to carry out their necessary mobile phone use in their daily lives (for contact with family or study needs) because these variables have been shown by previous studies to be related to individual mobile phone use (Butt and Phillips, 2008; Hong et al., 2012).

### Data analysis

SPSS 26.0 software was used for the internal consistency, descriptive statistics, correlations among the variables and regression analysis. Taking Neroticism as the antecedent variable, Self-emotional assessment as the intermediary variable, and Mobile phone use as the outcome variable, after adding the control variables, a hierarchical regression analysis was conducted to test hypotheses H1–H3. In addition, for the masking effect problems, we used the SPSS macro PROCESS developed by Hayes (2013), added control variables, and performed Bootstrap sampling to repeatedly extract 1,000 samples to calculate the 95% confidence interval (CI). We then observed whether the CI of each path included zero and gauged whether the masking effect was significant. Similarly, the PROCESS macro was used to test the moderating effect of love status. The subjects were divided into in/not in love groups to estimate the influence of self-emotional assessment on mobile phone use and the masking effect of self-emotional assessment at the different love status to test hypotheses H4 and H5.

## Results

### Common method bias test

As all of our sample data were collected by way of self-report, it was possible that they were affected by common method bias. Therefore, we carried out Harman’s single-factor test to assess this bias (Tang and Wen, 2020). In our study, based on the eigenvalue greater than 1, a total of 9 factors were extracted, and the 9 factors together explained 82.429% of the total variance of the questionnaire items, among which factor 1 explained 27.458% of the total variance, which was less than 40% of the critical threshold. This indicated that there was no serious common method bias in this study.

### Descriptive statistics

Table 1 presents the means and standard deviations of the study variables, and correlations between each of the variables, which provide preliminary support for subsequent hypothesis testing.

**Table 1:** Descriptive statistics and correlations among all variables.

	*M*	SD	1	2	3	4
1. Mobile phone use	3.00	0.55	1			
2. Self-emotional assessment	3.59	0.62	0.082*	1		
3. Neuroticism	14.13	5.05	0.242**	−0.155**	1	
4. Love status	0.41	0.49	−0.006	0.027	−0.080*	1

*n* = 896, for in love = 1, not in love = 0.

* *p* < 0.05;

** *p*< 0.01.

### Mediating effect testing

Following Baron and Kenny (1986), we used three steps to examine the relationship between neuroticism and self-emotional assessment, the relationship between neuroticism and mobile phone use, as well as the masking effect of self-emotional assessment on the relationship between mobile phone use and neuroticism.

In the first step, we used neuroticism as the independent variable and self-emotional assessment as the dependent variable. The results of the path analysis showed that neuroticism had a significant negative effect on self-emotional assessment (*b* = −0.012, *p* < 0.01).

In the second step, we took neuroticism as the independent variable and mobile phone use as the dependent variable. The results of the path analysis showed that neuroticism had a significant positive effect on mobile phone use (*b* = 0.026, *p* < 0.001).

In the third step, we took neuroticism and self-emotional assessment as the independent variables and mobile phone use as the dependent variable. The results of the path analysis showed that self-emotional assessment had a significant positive effect on mobile phone use (*b* = 0.136, *p* < 0.001), while neuroticism also had a significant positive effect on mobile phone use (*b* = 0.027, *p* < 0.001). This indicated that self-emotional assessment had a significant masking effect on the relationship between neuroticism and mobile phone use among college students.

The specific results are shown in Table 2.

**Table 2:** Hierarchical regression analysis with control variables.

Dependent variable	Self-emotional assessment		Mobile phone use		
	Model 1	Model 2	Model 3	Model 4	Model 5
**Control variable**					
Gender	−0.129**	−0.108***	0.251***	0.207***	0.222***
Grade	0.03	0.031**	−0.064**	−0.066**	−0.071***
Love status	0.02	0.01	0.025	0.046	0.045
Only child	−0.049	−0.05	−0.023	−0.021	−0.014
Area of source	0.035	0.04	0.077	0.067	0.061
Time spent on necessary mobile phone use	0.102*	0.099	0.025	0.032	0.018
Extraversion	0.008	0.006	−0.005	−0.001	−0.002
Psychoticism	−0.046**	−0.041***	0.014	0.004	0.009
**Independent variable**					
Neuroticism		−0.012***		0.026***	0.027***
Self-emotional assessment					0.136***
*R* ^2^	0.055	0.063	0.055	0.099	0.117
*F*	7.281***	7.485***	6.248***	10.501***	11.420***

* *p* < 0.05;

** *p* < 0.01;

*** *p* < 0.001.

In addition, we conducted a bootstrap test to verify the mediating effect of self-emotional assessment using the percentage-point bootstrap method for deviation correction proposed by Fang et al. (2012). We used the PROCESS macro in SPSS compiled by Hayes (2013) to perform a 1,000-sample analysis with a 95% confidence level. The results are shown in Table 3. The value of the total effect of neuroticism on college students’ mobile phone use was 0.026, with a confidence interval of [0.018, 0.035]. The direct effect value was 0.028, with a confidence interval of [0.020, 0.037]. The indirect effect value, given by the difference between the total effect and the direct effect, was −0.002, with a confidence interval of [−0.005, −0.001] excluding 0. This provided further proof that self-emotional assessment had a mediating effect on the relationship between neuroticism and mobile phone use. According to Wen and Ye (2014), due to the indirect and direct effects having opposite signs, so we concluded it as masking effect, the masking effect|ab/c| = 7.1%. Hence, the masking effect of self-emotional assessment on the relationship between neuroticism and mobile phone use was verified.

**Table 3:** Bootsrapping mediating effect analysis.

	Estimated effect	S.E.	Bootstrap 95% confidence interval	
			Lower bounds	Upper bounds
Total effect	0.026	0.004	0.018	0.035
Direct effect	0.028	0.004	0.020	0.037
Indirect effect	−0.002	0.001	−0.005	−0.001

Therefore, Hypotheses 1–3 were supported.

### Moderating effect testing

In Hypotheses 4 and 5, we predict that love status will moderates the relationship between self-emotional assessment and mobile phone use as well as the whole mediating effect mechanism. Since our moderating variable is a dichotomous variable, we coded the moderating variable (love status) 0–1 in advance (0 = not in love, 1 = in love), and then used the PROCESS macro Model 14 (Hayes, 2013) for moderation analysis. It can be seen from Table 4 that the interaction between love status and self-emotional assessment has a significant impact on mobile phone use (*b* = 0.15, *p* < 0.05).

**Table 4:** Moderating effect analysis.

	Estimated effect	S.E.	*t*	*p*	Bootstrap 95% confidence interval	
					Lower bounds	Upper bounds
Self-emotional assessment	0.12	0.04	3.51	0.00	0.05	0.19
Love status	0.12	0.04	2.62	0.01	0.03	0.20
Self-emotional assessment × Love status	0.15	0.07	2.11	0.03	0.01	0.30

**Figure 2:**
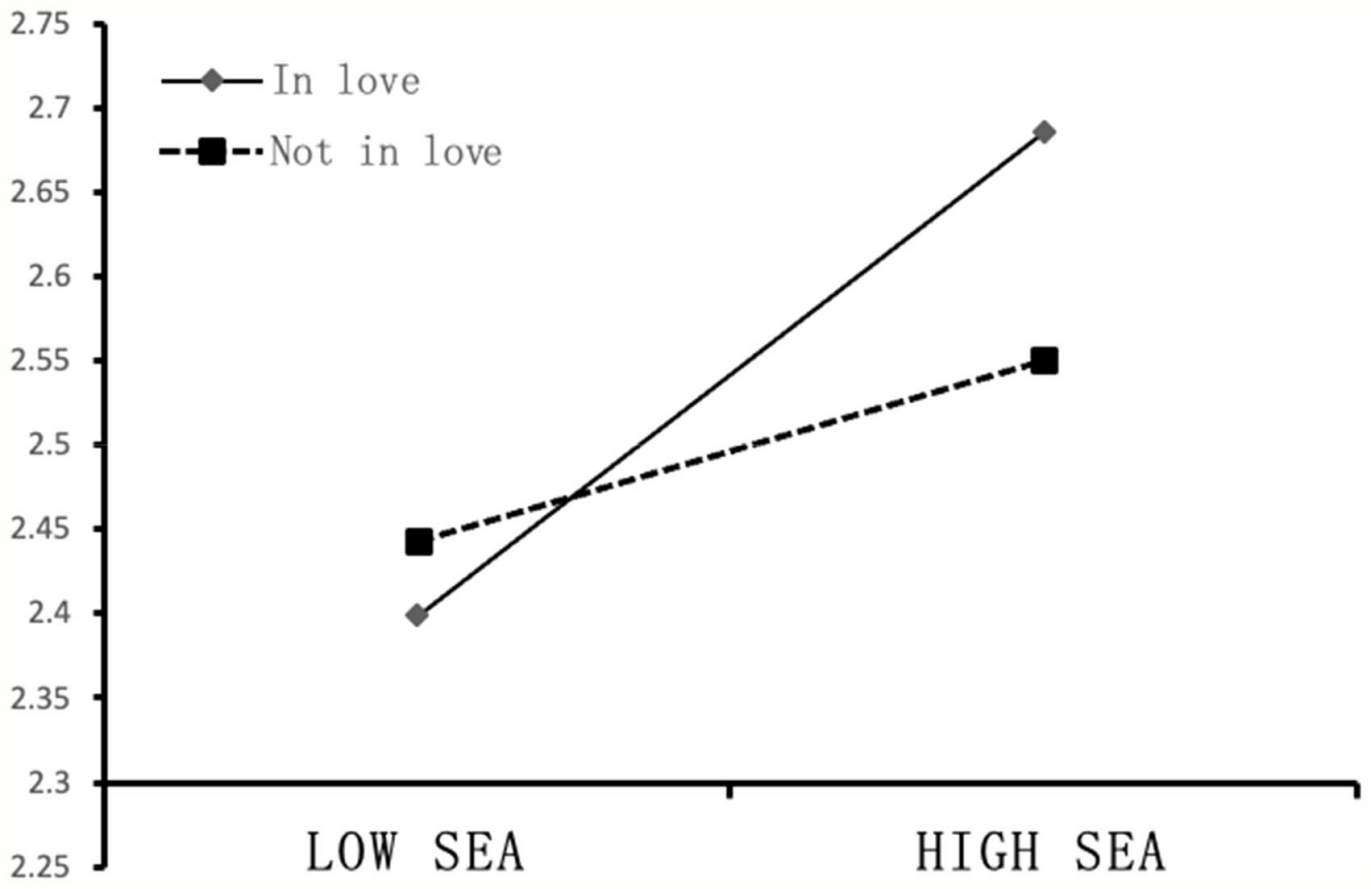
Moderating effect of love status.

Furthermore, we conducted a simple slope analysis and plotted the effect of negative emotions on rumination separately for students in love and not in love (Figure 2). When college students were not in love, there was a weak positive correlation between self-emotional assessment and mobile phone use (*b*_simple_ = 0.086, *p* < 0.05). When college students were in love, there was a relatively strong positive correlation between self-emotional assessment and mobile phone use (*b*_simple_ = 0.231, *p* < 0.001). These results indicates that compared with college students who are not in love, self-emotional assessment had a greater impact on the degree of mobile phone use of college students in love. Therefore, H4 was supported.

H5 predicted that the masking effect of self-emotional assessment on the relationship between neuroticism and mobile phone use is stronger when students are in love. The test results are shown in Table 5, the masking effect was stronger when students were in love [*ρ* = 0.0039, 95% CI = (−0.0081, −0.0013)] than when they were not in love [*ρ* = 0.0011, 95% CI = (−0.0039, 0.0006)]. The difference in strength between these two effects was significant [∆*ρ* = 0.0028, 95%, CI = (−0.0074, −0.0001)]. Therefore, H5 was supported.

**Table 5:** Moderated mediating effect analysis.

	Estimated effect	S.E.	Bootstrap 95% confidence interval	
			Lower bounds	Upper bounds
Not in love	−0.0011	0.0011	−0.0039	0.0006
In love	−0.0039	0.0017	−0.0081	−0.0013
Difference	0.0028	0.0017	−0.0074	−0.0001

Table 6 shows the data analysis results of each hypothesis, from which it can be clearly seen that H1–H5 were supported.

**Table 6:** Statistical analysis results.

Hypothesis	Path	[LLCI, ULCI]	Value of *p*	Decision
H1	N → MPU	[0.001, 0.046]	0.03	Support
H2	N → SEA	[−0.026, −0.009]	0.00	Support
H3	N → SEA → MPU	[0.067, 0.191]	0.00	Support
H4	Love × SEA → MPU	[0.148, 0.283]	0.00	Support
H5	N → Love × SEA → MPU	[−0.0074, −0.0001]	0.00	Support

The 95% CI and value of *p* reported in H1–H4 are the effect value indicators among variables, and in H5 are the difference value indicators between groups under different love states.

## Discussion

From the perspective of emotion, our study analyzed the internal mechanism of the influence of neuroticism on college students’ mobile phone use and the moderating effect of love status on this mechanism. Through the analysis, we found that neuroticism is an important factor affecting mobile phone use, college students with neurotic personalities tend to be highly susceptible to pressure and experience negative conditions such as social anxiety, depression, and excessive tension, such individuals then usually alleviate their negative emotions and achieve psychological compensation through using social media applications or playing entertainment games on their mobile phones. This also verifies the conclusions of previous studies (Liu and Campbell, 2017; Carvalho et al., 2018; Lei et al., 2020; Bowden-Green et al., 2021).

In addition, we also found that the self-emotional assessment is also an important predictor of mobile phone use. After individuals perceive and recognize their emotions, for the purpose of expressing or relieving emotions, they may use their mobile phones to communicate with others or use the entertainment and social media applications on their phones to escape from in-person reality temporarily. As shown in Model 5 in Table 2, Our results showed that when we controlled for the mediating variable (i.e., self-emotional assessment), the effect of neuroticism on mobile phone use increased, suggesting that self-emotional assessment did not facilitate, but partially masked the effect of neuroticism on mobile phone use. This may because individuals with high neuroticism often have a low level of self-emotional assessment, which renders them unable to accurately assess their own emotions. As a result, they cannot effectively use external tools (such as mobile phones) to deal with problems in life, which therefore reduces their use of mobile phones.

Third, As shown in Figure 2, our study found that love status plays a moderating role in the relationship between self-emotional assessment and mobile phone use. Individuals in love have high emotional sensitivity and greater emotional fluctuations (Yu, 2021), which means the level of self-emotional assessment is very important to them. Self-emotional assessment level affects how individuals decode and recognize their emotions, and then affect the decisions and future actions of individuals, so a higher level of self-emotional assessment can help them to identify and understand their own emotions, and reasonable use of external tools (such as mobile phone) to express release emotion. By contrast, students who are not in love have relatively small emotional ups and downs, which causes the level of their self-emotional assessment ability to have little impact on their decisions and behaviors. Therefore, self-emotional assessment has no significant impact on mobile phone use for these students.

Finally, we also found that love status moderated the whole covering mechanism of “neuroticism–self-emotional assessment–mobile phone use,” as shown in Table 6, only when the individual is in love, the masking effect of self-emotional assessment is significant. This also further demonstrates the importance of self-emotional assessment for individuals in love. For neurotic individuals in love, the stronger the self-emotional assessment ability, the stronger the masking effect on the relationship between neuroticism and mobile phone use, the smaller the direct effect of neuroticism on mobile phone use. By the same token, this may be because the emotional sensitivity of individuals in love is stronger, which causes their self-emotional assessment ability to have a more significant masking effect on the main effect.

### Theoretical implications

Our research makes several contributions to theory. First, our study provides insight into new factors affecting mobile phone use, namely self-emotional assessment. Through regression analysis, we confirmed that self-emotional assessment was positively correlated with the degree of mobile phone use for our participants. This may be because after individuals correctly perceive their own emotions, they become more adept at solving problems or expressing their emotions through external tools (e.g., mobile phones), which causes them to use mobile phones more frequently. Furthermore, our study confirmed the positive predictive effect of neuroticism on college students’ mobile phone use. Our finding is consistent with those of previous studies (Liu and Campbell, 2017; Carvalho et al., 2018; Lei et al., 2020; Bowden-Green et al., 2021). These findings deepen our understanding of mobile phone use among college students.

Moreover, our study enriches the existing theoretical framework explaining mobile phone use by further illustrating the mechanisms by which neuroticism influences mobile phone use. Although previous studies on the influence mechanism of the “neuroticism–mobile phone use” model have begun to take shape (Jiang et al., 2016; Marino et al., 2016; Zhang et al., 2020; Liu et al., 2020a). To the best of our knowledge, although emotional factors have been acknowledged to significantly affect mobile phone use (Gao and Chen, 2006; Volungis et al., 2020) their role has often been ignored in studies investigating the mechanism by which neuroticism influences mobile phone use. As an exception, Zhang et al. (2017) considered the mediating role of negative emotions, but no studies have focused on emotional cognition or the generation of emotional experience. Whether an individual can correctly perceive and explain their emotions determine their subsequent emotional experience, and thus the way in which they express their emotions. From the perspective of emotional cognition, this study examined the mediating effect of self-emotional assessment, and the masking effect is only significant for individuals in love, but not significant for individuals not in love.These findings further expand our understanding of the mechanisms of “neuroticism–mobile phone use,” provide new perspectives to explain mobile phone use among college students.

Finally, our study expanded the understanding of the boundary conditions delimiting the effect of neuroticism on mobile phone use. The results showed that the love status of college students moderated the relationship between their self-emotional assessment and mobile phone use. Specifically, compared with single college students, the self-emotional assessment level of college students in love has a more significant positive impact on mobile phone use. Moreover, love status in our study also moderated the process through which self-emotional assessment masked the effect of neuroticism on mobile phone use. When college students were in love, the masking effect was significant; when college students were not in love, the masking effect was not significant. These findings further expand our understanding of neuroticism and mobile phone use, provide new insights into factors influencing mobile phone use among contemporary college students, and increase our understanding of the differentiation of mobile phone use phenomena among different college students.

### Practical implications

Our study provides several practical implications for societies and schools. First, our study found that individuals with higher levels of self-emotional assessment used mobile phones to a greater extent. Individuals with high levels of self-emotional assessment can readily perceive their emotions and find appropriate ways to express them. Mobile phones provide a channel for college students to release their emotions and have gradually become an important tool that they can use to solve problems. We believe that schools and parents should not excessively curb students’ use of mobile phones. Although previous studies have shown that the frequent use of mobile phones can cause a range of negative conditions (Kumar and Anupama, 2019; Jacob, 2020; Liu et al., 2020b), mobile phone use may also act as a kind of emotional sedative for some people (especially for neurotic individuals). It can be a tranquilizer that allows people to temporarily forget their worries. Meanwhile, Jiang et al. (2016) found that mobile phone network information services (search engines, online news platforms, etc.) were the most popular and frequently used mobile phone network services among college students. College students searched for information using their mobile phones to help them answer questions encountered in their studies and lives. Therefore, we believe that college students can improve their self-emotional assessment ability of emotions through some intervention measures, thus increasing individuals’ correct perception and expression of emotions, so that they can make better use of external tools such as mobile phones ability and thus better express their emotions and solve problems in their studies and lives.

Second, in line with previous studies, we found that college students with higher neuroticism used mobile phones more frequently. Neurotic individuals have poor emotion regulation ability and often experience negative conditions such as anxiety and depression. Because the social media and entertainment functions of mobile phones can meet the psychological needs of neurotic individuals, mobile phones become the best tool with which they can eliminate their negative emotions. We therefore advocate the rational use of mobile phones for neurotic students to better express their emotions and solve problems. On the other hand, it is also necessary to control the time of mobile phone use, improve self-control, avoid a series of bad performances such as self-isolation and sub-health caused by mobile phone dependence, and guard against addiction.

Third, we found that love status moderated the relationship between self-emotional assessment and mobile phone use, as well as the process through which self-emotional assessment masked the effect of neuroticism on mobile phone use. These results indicate that the mobile phone use of neurotic individuals in love is more likely to be influenced by self-emotional assessment. Therefore, parents and schools should pay more attention to psychological counseling of neurotic individuals in love and provide timely and targeted guidance, help them relieve tension and anxiety, and face their emotions honestly and courageously, increase their effective use of mobile phones so that they can better identify and manage their emotions.

### Limitations and future research

This study inevitably had some limitations. First, our participants were recruited from only Chinese universities. The sample selection range could be expanded in the future to increase the universality and reliability of the results. Second, the datas in our study are entirely self-reported, which increases the chance of common method bias. Future research can try to collect data from multiple information sources to make the test results of the model more convincing. Third, we only collected cross-sectional data. Future research could measure different variables at different time points to further reduce the influence of common method bias on the results and to increase the convincingness of the causal relationships. Finally, this study only investigated self-emotional assessment as the mediating variable. Future research could introduce other variables such as social identity and emotional regulation strategies to explore their influence on mobile phone use, or their correlation with mobile phone use. This would enrich the research on mobile phone use, and contribute toward a more comprehensive understanding of college students’ mobile phone use.

## Conclusion

Focusing on emotion, we analyzed the mechanism by which neuroticism influences college students’ mobile phone use and found that self-emotional assessment masks the relationship between neuroticism and mobile phone use. Furthermore, to account for individual differences in self-emotional assessment ability, we explored the moderating effect of love status on the relationship between self-emotional assessment and mobile phone use.

## Data availability statement

The original contributions presented in the study are included in the article/supplementary material, further inquiries can be directed to the corresponding author.

## Author contributions

WC designed and drafted this work. XW participated in the paper writing and analyzed data. SS, QL, and ZG has made a substantial, direct, and intellectual contribution to the work. All authors contributed to the article and approved the submitted version.

## Funding

This work is supported by the Foundation for Philosophy and Social Sciences Researh Project of Department of Education of Hubei Province (21Y002) and MOE (Ministry of Education in China) Project of Humanities and Social Sciences (18YJC190021).

## Conflict of interest

The authors declare that the research was conducted in the absence of any commercial or financial relationships that could be construed as a potential conflict of interest.

## Publisher’s note

All claims expressed in this article are solely those of the authors and do not necessarily represent those of their affiliated organizations, or those of the publisher, the editors and the reviewers. Any product that may be evaluated in this article, or claim that may be made by its manufacturer, is not guaranteed or endorsed by the publisher.
